# Editorial for the Special Issue “Current Research on Cancer Biology and Therapeutics: Fourth Edition”

**DOI:** 10.3390/ijms27198568

**Published:** 2026-09-25

**Authors:** Maruan Hijazi, Rafael Coveñas

**Affiliations:** 1 Group USAL-BMD (Bases Moleculares del Desarrollo), University of Salamanca, 37007 Salamanca, Spain; hijazivm@usal.es; 2 Institute of Neuroscience of Castilla and León (INCYL), iBRAINS-IN-CYL, 37007 Salamanca, Spain; 3 Institute of Biomedical Research of Salamanca (IBSAL), 37007 Salamanca, Spain; 4 Laboratory of Neuroanatomy of the Peptidergic Systems, Institute of Neuroscience of Castilla and León (INCYL), University of Salamanca, 37007 Salamanca, Spain

The fourth edition of this Special Issue, entitled Current Research on Cancer Biology and Therapeutics, includes twelve papers (nine articles and three reviews). These papers are focused on oncolytic vaccinia virus [1], ovarian [2], endometrial [3], breast and colorectal tumors [4,5,6,7], periocular morphoeic basal cell, oral squamous cell and urothelial carcinomas [8,9,10], liposarcoma [11], and glioblastoma [12].

Oncolytic virotherapy is a promising strategy to fight solid tumors; however, single-agent treatments are often limited by inadequate tumor lysis and insufficient activation of the immune system [1]. The group led by Dr. Olga N. Alekseeva and Pavel O. Vorobyev (Engelhard Institute of Molecular Biology, Russian Academy of Science, Moscow, Russia) reported, in an aggressive murine mammary adenocarcinoma model, a combined therapy using two recombinant alternatives of the oncolytic vaccinia virus, armed with either the interleukin 15 receptor subunit alpha (VV-mIL15Rα) or the herpes simplex virus thymidine kinase (VV-HSVtk), in order to increase immune stimulation and cytotoxicity [1]. In in vitro experiments, VV-HSVtk showed a dose-dependent cytotoxicity increased by ganciclovir via HSVtk-mediated phosphorylation into a cytotoxic nucleoside analog, and the co-culture of donor-derived NK cells and VV-infected cancer cells further augmented the oncolytic effectiveness, whereas, in in vivo experiments, the combined treatment (VV-HSVtk, VVmIL15Rα and ganciclovir) reverted the tumor and prolonged survival, an outcome that neither component achieved as monotherapy [1]. An increased infiltration of lymphocytes in the tumor sites, and no splenic and liver toxicity, were observed. In sum, this study supports that combining direct viral cytotoxicity, an HSVtk/ganciclovir-mediated suicide gene approach, and interleukine-15-pathway-targeted immunomodulation within an oncolytic vaccinia platform could greatly ameliorate the antitumor effects in an experimental breast cancer model. This study supports the development of multimodal armed vaccinia virus platforms in which immunostimulatory transgene expression and cytotoxicity are planned to act together.

The group led by Dr. Pallavi Nayak and Michela Varani (Faculty of Medicine and Psychology, University Hospital Sant’Andrea, Sapienza University of Rome, Roma, Italy) reviewed the involvement of the luteinizing hormone-releasing hormone (LH-RH), also known as gonadotropin-releasing hormone, in ovarian cancer [2]. LH-RH receptors are expressed in ovarian tumors (around 80% of cases) suggesting the use of further effective and specific antitumor strategies (e.g., targeted drug delivery). This is the main goal of this narrative review: to show the advances in and development of LH-RH receptor-targeted strategies to fight ovarian cancer, one of the most lethal gynecologic malignancies. This review includes LH-RH–conjugate nanosystems and LH-RH–peptide drug delivery systems and covers in vitro studies, preclinical models and ongoing clinical trials. The authors stated that the results demonstrated greater preclinical efficacy, achieving tumor growth inhibition of between 50% and 80% and cell uptake between two and four times greater, in addition to lower systemic toxicity, whereas clinical studies showed a response/disease control rate of about 50%, supporting better tumor accumulation of drugs, small interfering RNA, and diagnostic agents. Accordingly, targeting LH-RH receptors is a promising specific approach to combat ovarian cancer and to increase therapeutic outcomes. In sum, this review shows the enormous potential of LH-RH–conjugate nanosystems and LH-RH–peptide drug delivery systems to treat ovarian cancer, either alone or in combination with current antitumor strategies used in clinical practice. Moreover, this review is a clear example of the importance of the peptidergic systems as antitumor targets as well as diagnostic markers due to the overexpression of peptide receptors in cancer cells.

Endometrial cancer is a frequent gynecologic malignancy showing increasing incidence due to continued exposure to unrestricted estrogens, obesity, and reproductive causes [3]. On many occasions, hereditary endometrial cancer remains underdiagnosed, and this means that a crucial time is lost for its detection and treatment. The Hanna Markiewicz group (Faculty of Medicine, Medical University of Warsaw, Warsaw, Poland) has reviewed this disease [3]; hence, this review is focused on the hereditary predisposition to develop endometrial cancer, particularly Lynch syndrome, resulting from germline mutations in DNA mismatch repair genes and being associated with an increased risk of developing endometrial cancer, reaching up to 71% in those individuals with *MutS* homolog 6 mutations. In this review, inheritance patterns, the genetic mechanisms involved, the diagnostic strategies, and the clinical implications of genetic counseling, screening and optimization of the treatment are shown. Importantly, in addition to Lynch syndrome, the authors focus this review on rarer genetic conditions that contribute to the risk of developing hereditary endometrial cancer, such as hereditary breast and ovarian cancer syndromes, polymerase proofreading-associated polyposis, Li–Fraumeni syndrome and Cowden syndrome. All the scientific knowledge gathered in this review is crucial for the development of improved preventive and therapeutic approaches for a better and more accurate diagnosis and for an improved personalized monitoring of patients with hereditary endometrial cancer.

Breast cancer research is increasingly characterized by multi-omic investigations that uncover crucial transcriptional signatures, metabolic pathways, and microenvironmental dynamics. Analyzing developmental regulators, Scimeca et al. (Department of Experimental Medicine, University of Rome “Tor Vergata”, Rome, Italy) [4] demonstrated that the paired-box (PAX) family of transcription factors are critically reactivated in primary breast carcinomas and directly correlate with key oncogenic hallmarks, including epithelial–mesenchymal transition (EMT), immune evasion, and cell death [4]. Notably, PAX1 and PAX9 positively correlate with apoptotic and cell-fate mediators such as BAX and BARD1 [4]. Concurrently, PAX3, PAX5, and PAX8 demonstrate strong positive associations with immune checkpoint markers like PD-L1 and TIGIT, while a transcriptomic PAX2–PAX7 signature predicts lymph node metastasis with high accuracy [4]. Complementing these genomic profiles, Masisi et al. (School of Medicine, University of Nottingham, Nottingham, United Kingdom) [5] evaluated metabolic vulnerabilities in luminal breast cancer, establishing that the kidney-type glutaminase isoform GLS is essential for tumor survival [5]. The genetic knockdown of *GLS* significantly reduces cell proliferation and induces apoptosis while arresting cells in the G1 phase, whereas pharmacological inhibition with the allosteric inhibitor CB-839 (Calithera Biosciences Inc., San Francisco, CA, USA) shows minimal efficacy in luminal subtypes due to metabolic compensation, highlighting the therapeutic differences between complete physical protein removal and enzymatic inhibition [5]. This contrast underlines why understanding metabolic dependencies is critical when evaluating glutaminase-targeted therapeutics in clinical settings [13]. To target the therapeutic vulnerabilities of the tumor microenvironment (TME) in triple-negative breast cancer (TNBC), Banqueri-Pegalajar et al. (Department of Molecular Biology and Biochemistry, Faculty of Science, University Málaga, Málaga, Spain) investigated the bioactive ellagitannin punicalin (Merck, Darmstadt, Germany) [6]. Punicalin selectively modulates redox homeostasis and autophagy in a cell-type-specific manner [6]. While it exerts a robust antioxidant response in TNBC cells by reducing basal and hydrogen-peroxide-induced ROS, it impairs stress-adaptive autophagy and induces a pro-oxidant state in tumor-associated microvascular endothelial cells (HMEC-1) [6]. Functionally, this selective disruption translates into impaired cell migration and compromised endothelial tube formation under rigid, tumor-like matrix conditions without affecting normal endothelium, validating the therapeutic strategy of disrupting microenvironmental cell communication and vascular sprouting [14].

The therapeutic modulation of inflammatory signaling cascade pathways represents a pivotal strategy to halt colitis-associated colorectal cancer (CRC). Robinson et al. (The University of Texas MD Anderson Cancer Center, Houston, USA) evaluated the therapeutic efficacy of TTI-101 (50 mg/Kg formulated in Labrasol 60%; Gatte Fosse, Saint-Priest, France/PEG-400 40%; Spectrum Chemicals, New Brunswick, NJ, USA), a selective small-molecule inhibitor targeting the phosphotyrosyl peptide-binding pocket within the STAT3 SH2 domain [7]. In an azoxymethane plus disodium sulfate mouse model of chronic colitis-induced CRC, daily oral administration of TTI-101 reduced colonic adenoma numbers by 89%. This marked therapeutic benefit directly correlated with the suppression of nuclear pY-STAT3 levels in both epithelial and stromal compartments of dysplastic colonic mucosa [7]. Furthermore, transcriptomic analysis demonstrated that TTI-101 normalized the aberrant colonic transcriptome by downregulating a subset of pro-oncogenic genes co-targeted by STAT3 and STAT1 (such as MMP7, S100A9, TIMP1, IL1B, and CCL2) associated with tumor progression and metastasis [7]. Given the robust connection between chronic barrier disruption, intestinal inflammation, and elevated malignancy risk [15], these findings highlight the clinical utility of targeting the STAT signaling pathway, particularly as TTI-101 has shown promising target engagement and safety in advanced solid tumor clinical trials [16].

In periocular basal cell carcinoma (BCC), multi-omic profiling has unveiled the molecular heterogeneity driving locally invasive morphoeic BCC (mBCC) versus more indolent nodular BCC (nodBCC) subtypes. Bladen et al. (Kings College Hospital, London, United Kingdom) identified subtype-specific somatic driver mutations, demonstrating that loss-of-function variants of the potential tumor-suppressors filamin B (*FLNB*) and *HECTD4* define the mBCC phenotype [8]. *FLNB* mutations specifically cluster in the Filamin domain 24, which would potentially disrupt its interaction with FLNA2, and correlate with a significant downregulation of *FLNB* expression in mBCC tumors [8]. Reconstituting this genetic environment in human NEB1 keratinocytes through a double-*PTCH1*/*FLNB* knockdown mimetics mBCC biology in vitro, resulting in colony disorganization, enhanced cell migration, and the recruitment of active stromal fibroblasts [8]. Moreover, mBCC is characterized by a GLI2-dominant, aberrant activation of the hedgehog pathway across both the tumor nest and the surrounding fibrotic stroma, which contrasts with the canonical, physiologically active hedgehog levels seen in nodular tumors [8]. Comparative clinicopathological analyses confirm that high-risk periocular BCC subtypes present unique molecular features compared to sporadic nodular lesions and basal cell nevus syndrome [17].

The mechanical properties of the extracellular matrix dynamically regulate cellular plasticity and therapeutic resistance in head and neck squamous cell carcinoma (HNSCC). Buonvino et al. (Department of Chemical Science and Technologies, University of Rome “Tor Vergata”, Rome, Italy) investigated the impact of microenvironmental stiffness on oral squamous cell carcinoma cells using 3D PEG–silk fibroin hydrogel tumoroids [9]. Treatment with the peptide CIGB300 (Center of Genetic Engineering and Biotechnology of Havana, Cuba), a pro-apoptotic therapeutic that binds to nucleophosmin (B23/NPM1) and halts its CK2-mediated phosphorylation [18], induced significantly higher cell death under high-matrix-rigidity conditions (7.81 kPa, mimicking invasive tumor stiffness) compared to soft environments [9]. Interestingly, spectroscopic and diffusion analysis revealed that while FITC-CIGB300 diffusion kinetics were slower in stiffer matrices, tumoroid cells adapted to the rigid environment by downregulating B23/NPM1 expression and the multidrug resistance ABC transporter P-glycoprotein, thereby sensitizing cells to the peptide’s cytotoxicity [9]. These insights highlight the therapeutic importance of matrix rheology as a synergistic factor that shapes tumor drug sensitivity.

Patrichi et al. (Romanian Academy of Medical Sciences, Bucuresti, Romania) quantified the relative gene expression of *SOX9*, *GATA3*, and *GATA4* across diverse histological subtypes of liposarcomas, observing consistent transcriptional upregulation in a majority of malignant specimens [11]. The highest expression levels of *SOX9* and *GATA4* occurred in atypical lipomatous tumor/well-differentiated liposarcoma (ALT/WDLS) cases, suggesting that the reactivation of these developmental regulators represents early events in liposarcomagenesis [11]. In silico network modeling revealed that SOX9 and GATA4 are embedded within broad transcriptional circuits driven by RELA, MYC, and NANOG, and are targeted by a common signature of microRNAs regulating EMT and stemness, while GATA3 and GATA4 display a robust inter-gene correlation [11]. Updated molecular classifications emphasize the necessity of integrating gene expression profiles to refine soft-tissue sarcoma diagnosis [19].

In their comprehensive review, Juanes-Gusano et al. (Institute of Neuroscience of Castilla and León, iBRAINS-IN-CyL, University of Salamanca, Salamanca, Spain) [12] evaluate the therapeutic limitations and medicinal chemistry advancements of small-molecule kinase inhibitors in glioblastoma. While these compounds represent a compelling strategy, their clinical efficacy is severely hindered by the blood–brain barrier and active efflux transporters like P-glycoprotein and breast cancer-resistance protein. To overcome these pharmacokinetics bottlenecks, the authors propose next-generation medicinal chemistry approaches to reduce transporter recognition and enhance passive diffusion. These strategies include macrocyclization, structural rigidification, lowering the topological polar surface area, and bioisosteric capping of polar handles. Alternatively, carrier-mediated prodrugs targeting transporters like LAT1 provide a viable rescue strategy to facilitate blood–brain barrier translocation of potent scaffolds. Furthermore, Juanes-Gusano et al. [12] analyze clinical failures (e.g., paxalisib and osimertinib), demonstrating that single-node monotherapies inevitably fail due to glioblastoma’s hallmark clonal heterogeneity and compensatory pathway hyperactivation. To suppress tumor adaptation, they advocate for multi-targeted agents or rational dual-node combinatorial regimens (such as co-targeting PI3K/mTOR and MAPK/ERK pathways). Synergistic networks can also be established by combining kinase inhibitors with DNA damage repair inhibitors, tumor-associated macrophage re-education, or physical barrier disruption via MR-guided focused ultrasound. Lastly, they emphasize that bridging the translational gap requires replacing genetically drifted, serum-cultured cell lines with patient-derived glioma stem cell spheres and orthotopic xenografts to better recapitulate the cellular architecture of the disease.

The group led by Dr. Anait S. Levenson (Lewyt College of Veterinary Medicine, Long Island University, Brookville, USA) studied the molecular processes and functional roles of the oncogenic transcriptional regulator named metastasis-associated protein 1 in the canine urothelial carcinoma [10]. The involvement of this protein in epithelial-to-mesenchymal transition and its interaction with the BRAF signaling were studied. The overexpression of metastasis-associated protein 1 occurs in urothelial carcinoma, and it has been related to aggressive clinicopathological features. The silencing of this protein blocked the survival, proliferation and migration of urothelial carcinoma cell lines, inhibited xenograft tumor growth and reduced mutant BRAF expression in K9TCC-PU-AxA cells but increased wild-type BRAF expression in K9TCC-PU-Sh cells, suggesting a context-dependent regulation of the BRAF signaling. Moreover, metastasis-associated protein 1 knockdown decreased COX2 and MTA2/3 expressions and promoted changes in epithelial-to-mesenchymal transition regulators (cyclin D1, slug, snail). This study demonstrated that metastasis-associated protein 1 regulated the progression of canine urothelial carcinoma, and hence this protein is a promising therapeutic target to treat this disease.

This is the fourth edition of this Special Issue, named Current Research on Cancer Biology and Therapeutics [20,21,22]. In addition to the articles published in the fourth edition, in the three previous editions a plethora of potential and promising anticancer strategies were presented, using podophyllotoxin–benzothiazole compounds, bispecific antibodies, bioactive peptides or peptide receptor antagonists, lidocaine combined with 5-fluorouracil or cisplatin, a combination of cisplatin with cannabinoid delta-9-tetrahydrocannabinol, benzo[a]phenoxazine derivatives, sarco/endoplasmic reticulum calcium ATPase inhibitors, acetylcorynoline, immune checkpoint inhibitors, 4-(dimethylamino) phenyl-5-oxopyrrolidine derivatives, poly-ADP ribose polymerase inhibitors and local anesthetics [20,21,22]. Moreover, antitumor approaches to combat neuroblastoma, glioma, melanoma, seminoma, teratoma, pancreatic ductal adenocarcinoma, papillary thyroid and hepatocellular carcinomas, and ovarian, bladder, prostate, breast and colorectal cancer were also reported in the three previous editions [20,21,22]. In sum, the four editions of this Special Issue (including 38 original articles, reviews and case reports) greatly increase the knowledge of cancer development and present a plethora of promising and different antitumor strategies, opening numerous lines of investigation with great potential for translational research.

## Data Availability

The original contributions presented in this study are included in the editorial. Further inquiries can be directed to the corresponding author.
